# Strategies to Reach Nomadic Children During Polio SIAs: Experience in Dadaab and Fafi Sub-Counties of Kenya after the 2013-2014 Polio Outbreak

**DOI:** 10.29245/2578-3009/2021/S2.1110

**Published:** 2021-04-12

**Authors:** Abdi H. Ahmed, Gedi Mohamed, Joseph Okeibunor, Iheoma Onuekwusi, Pascal Mkanda, Samuel Okiror

**Affiliations:** 1 WHO Nairobi Kenya; 2 WHO Regional Office for Africa (WHO AFRO), Brazzaville, Congo; 3 WHO Horn of Africa Coordination Office (HOA), Nairobi KENYA

## Abstract

**Background:**

Poliomyelitis, often called polio is a viral paralytic disease caused by Polioviruses. Although all susceptible individuals are at risk of getting infected, only about 1% become paralyzed. During the 2013 Polio Outbreak in Garissa County in Kenya, 50% of the confirmed cases were from the nomadic population although it comprises of only less than 20% of the total population in the county. Following concerns from the Horn of Africa Polio Technical Advisory Group (TAG) regarding inadequate vaccine coverage of nomadic population, several strategies were put in place to improve coverage and Acute Flaccid Paralysis case reporting among nomads in the rest of the planned 2014 polio vaccination campaigns. We describe strategies initiated from April 2014 by the Ministry of Health and partners to reach children in nomadic settlement in the two sub-counties of Dadaab and Fafi of Garissa County.

**Methods:**

The strategies involved improving the mapping and tracking of the nomadic population by establishing lists of nomadic settlements obtained from local clan leaders and government administrators, their <5-year-old populations and focal persons. Focal persons were used to mobilise residents in their respective settlements and guide vaccination teams during campaigns. Settlement leaders were sensitised to report cases of Acute Flaccid Paralysis. In remote hamlets, trained community health volunteers were used as vaccinators. In such places drugs for common illness were also provided during the campaigns. A tracking tool to monitor nomadic population movement and special tally sheets to capture data were created. Training of vaccination personnel and intense social mobilisation activities was done.

**Results and conclusion:**

About 2,000 additional children, from both nomadic and non-nomadic areas were reached when the new initiatives were started. For the first time, an actual number of nomadic children accessed was documented. Suspected AFP cases continued to be reported from nomadic settlements, and the number of zero dose children among the nonpolio AFP cases dropped. With modification and improvement, these strategies may be used to take health services such as routine immunisation to nomadic communities and reduce their vulnerability to vaccine preventable disease outbreaks.

## Introduction

A nomad is a member of a people or tribe who according to the changing seasons move from one place to another to meet the needs of their livestock. Movement is within a well-defined territory, but unlike pastoralists they generally do not form permanent homes in any one place^1^.

More than 60% of the nomads and semi-nomads in the world live in Africa^2^. Pastoralists migrate periodically with their herds to maximally exploit scarce pasture and water resources which they need for their animals and themselves. Mobility is an important determinant of health worldwide, and nomadic populations in sub-Saharan Africa are no exception^3^. Given their lifestyle, there is inadequate systematic surveillance data on the health status of nomads. In most of sub-Saharan Africa, access to health care services by nomads is poor compared to settled populations, and there is no satisfactory strategy devised yet to deliver proper health care to remote populations. Disease control programmes also often fail to reach migratory populations^4–7^. For example, a study among the settled and nomadic Rendille in Kenya revealed that all children >12 months of age in Korr village had full immunisation coverage, while among their nomadic counterparts immunisation coverage was 0%^8^. In East Africa, the nomadic Turkana population was found to suffer a substantially higher infant mortality than settled Turkana agriculturalists^9^.

Immunisation is one of the most successful and cost effective public health interventions. It has eradicated smallpox, lowered the global incidence of polio so far by 99% and achieved dramatic reductions in illness, disability and death from diphtheria, tetanus, whooping cough and measles^10^. Despite the efforts of international organisations and national governments, immunisation coverage rates, a major indicator of health service delivery, show that in sub-Saharan Africa, nomads are inadequately accessed by the conventional health system. They also form a pool of susceptible populations where an outbreak of communicable disease can occur at any moment^11^. Further, nomads can be active transmitters of disease to the communities into which they move. One of the major reasons why the world’s last case of smallpox occurred in Somalia was a continual movement of the nomads and their reintroduction of the disease into the settled populations. Moreover, diseases that are thought to be under control can remain in inaccessible pockets of infection among nomads. During migration, they may transport the disease over great distances and across national boundaries^12^.

The exact number of nomads in Kenya is not known. Apart from being highly mobile, other factors such as weak health systems, insecurity, long and unsecured porous border with Somalia, the presence of a large and highly mobile refugee population in the County and years of marginalisation have contributed to sub-optimal immunisation coverage in this part of the country. The effect has been frequent outbreaks of vaccine preventable diseases (VPDs) due to low coverage and therefore herd immunity in these hard to reach areas.

In April 2013, a polio outbreak was confirmed in Banadir, South Central Somalia and quickly spread across borders to the neighbouring Ethiopia and Kenya^11^. It led to >200 cases of polio, accounting for more than half of all confirmed polio cases in the world that year^12^. In May 2013, a wild poliovirus type 1 (WPV 1) outbreak was confirmed in a refugee camp in Dadaab, Garissa County in North Eastern Kenya bordering Somalia. Genetic sequencing results showed that the virus was closely linked to an outbreak in Somalia that was confirmed earlier in May 2013. By July 2013, 14 cases of WPV 1 were confirmed among refugees in Dadaab and host community in Dadaab, Fafi and Hulugho sub-Counties of Garissa County. Seven out of the 14 cases were reported from the host nomadic community which comprises <20% of the residents in the County. It is important to note that only, 1% of people infected with polio become paralysed, and for every paralysed child there are approximately 200 children infected and asymptomatic around it^13^. This issue is significant because the actual number of people infected by polio both within the refugee camps and host nomadic community was probably very large. Shortly after, the Somali region of Ethiopia also reported an outbreak of genetically related WPV 1^14^. Two earlier outbreaks in Dadaab Refugee Camps, a type 2 circulating vaccine derived polio virus (cVDPV2) in 2012 and WPV1 in 2006 were also importations from Somalia.

Despite these outbreaks in the past, no specific strategies were designed to reach children in the nomadic population either through the routine immunisation programme or during the polio vaccination campaigns. Micro-planning for supplementary immunization activities (SIAs) focused on reaching children within urban areas and known permanent settlements, campaign coverage data was not disaggregated to show the various population sections and coverage of nomadic children was not in the post-campaign reviews agenda. The lack of specific strategies was because the nomadic population in this area was not significantly affected by the other outbreaks before the 2013 outbreak. Further, outside the outbreak period, the participation of the nomadic community in Acute Flaccid Paralysis (AFP) surveillance, one of the four strategies for polio eradication was minimal. The Horn of Africa Polio Technical Advisory Group (HOA-TAG) also expressed concern at the quality of SIAs in these areas and recommended more focus on affected areas, including improving strategies to reach nomadic and other hard to reach populations^15^.

Initiating innovative and sustainable strategies to reach these populations was therefore discussed as a priority to enhance polio eradication measures and in response to the HOA Polio TAG call. Such measures were expected to reduce the number of under or unimmunised children in the area and therefore minimising the risk of re-importation of wild poliovirus. It was also intended to bring the nomadic community into the loop of the AFP Surveillance programme to improve case reporting. Subsequently, a number of strategies were put in different areas to improve coverage among nomads. The objective of this article is to describe those interventions in the two sub-Counties designed improve mapping and tracking of the nomadic population in the two sub-Counties to reach them with polio vaccines during campaigns. These two sub-Counties were selected because six out of the seven cases reported from the nomadic population were reported here.

## Methods

### Intervention area

The two sub-Counties of Dadaab and Fafi of Garissa in North eastern Kenya were identified for implementation of the new strategies. Both have long porous borders with Somalia to the East. They are host to a 300,000 - 400,000 refugee population, largely from Somalia and other Horn of Africa countries for > 20 years. Maintaining linkage to their home countries, the refugees return to the camps in Kenya after visiting their countries. Polio and other communicable diseases have been imported severally into the camps through these movement. The area is semi-arid, has poor infrastructure and security challenges. Each of the two sub-Counties is divided into three wards. To initiate the new strategies, consent was obtained from County and sub-County Ministry of Health officials, local tribal chiefs and respective government administrators.

### Target population

The target for the initiatives was the section of the population living in the two sub-Counties, who depending on the season, move from one location to another with their livestock and was estimated to be <20% of the combined total populations of 210,185 (Dadaab 87,880, Fafi 122,305) of the area. In 2016, the oral polio vaccine (OPV3) coverage in both sub-Counties was <80% (Dadaab 72%, Fafi 70%). Three out of the five polio outbreaks between 2006 and 2014 in Kenya were reported from here.

### New strategies to reach more nomadic children

Responding to the recommendations from the Horn of Africa TAG and as part of the outbreak vaccination response, the new initiatives were implemented in the two sub Counties (Table 1). These new strategies were intended to improve the vaccination coverage among nomadic children during polio SIAs and also establish the participation of the nomadic population in AFP surveillance. The initial step was to create a list of nomadic settlements in both sub-Counties and then from each such settlement identify a contact person. These contact persons were then linked to the Ministry of Health (MoH) polio campaign planning team. The second step was to sensitise settlement leaders on AFP case definition and reporting. During campaigns, MoH planning team would select specific teams to reach these settlements. Besides OPV, the selected teams will carry with them basic drugs to treat common childhood illnesses they may find in the field.

### Creating a list of settlements of nomadic hamlets

The nomads in this area live and travel in groups of families of a clan or sub-clan. Elders make most of the decisions touching on the lives of its members including movement and acceptance of government services. Two clans, Aulihan (in Dadaab sub-County) and Abdwak (in Fafi sub-County) live in this area. Lists of their settlements and possible watering points were to be prepared down to the lowest administrative units (sub-location) in the two subCounties to reach these families. Clan elders, water point and borehole operators and community health volunteers and government administrators in the two sub-counties were to be used for this purpose. The following information was collected for each settlement using a tracking form: location of the village, head of the family or village, the number of <5-year-old children in the settlement and available channels for social mobilisation. Before each round of polio vaccination campaign, this information on the number of settlements and their geographical locations was captured in the sub-County polio supplemental immunisation activities micro-plans.

A contact person with whom the MOH could easily communicate with was identified by members of every listed settlement. These contact persons, who were invariably male, were either living in the settlements or were family members living in trading or market centres within the area and were easy to contact by MoH when required. In a few settlements, the head of the family was also the contact person. Before each vaccination round, the contact person would be contacted to give information on the actual location of nomadic family and to participate in social mobilisation for the campaign. During the campaign, the contacts are expected to guide the vaccinators to the respective settlements, while those among them who were interested were recruited as volunteers in the campaign.

To reach children in settlements located far from main centres or where access was limited to motorised transport or by insecurity, community health volunteers (CHVs) drawn from the relevant clans and trained to give oral polio vaccine were sent. CHVs were also stationed at boreholes and some water points to administer the vaccine to children coming to these places with their parents after 4.00 pm when vaccinators closed work for the day.

All OPV SIA teams visiting nomadic settlements or watering points also carried with them drugs used for managing common childhood illness such as diarrhoea and acute respiratory infections.

All teams were provided with a special tally and summary sheets designed to capture children in nomadic settlements. At the end of each campaign, the number of children reached and their polio vaccination status was summarised by sub-County.

### Sensitising settlement leaders on AFP case definition and reporting

From the start of the outbreak, attempts were made to reach out to the nomadic community to involve them in reporting suspected AFP cases. These initiatives started 8 months after the last confirmed case (July 2013) and were intended to ensure the continued participation of the nomadic population in AFP Surveillance. Experienced CHVs were assigned to each listed nomadic settlement in the two sub-counties to sensitise the identified leaders and contact persons on the lay case definition, and how to report a suspected AFP cases. Any suspected AFP case was to be reported to the nearest health facility.

Data for this article are derived from the 2013-2015 polio supplementary immunisation campaign coverage and AFP Surveillance database.

## Results

**Figure 1:**
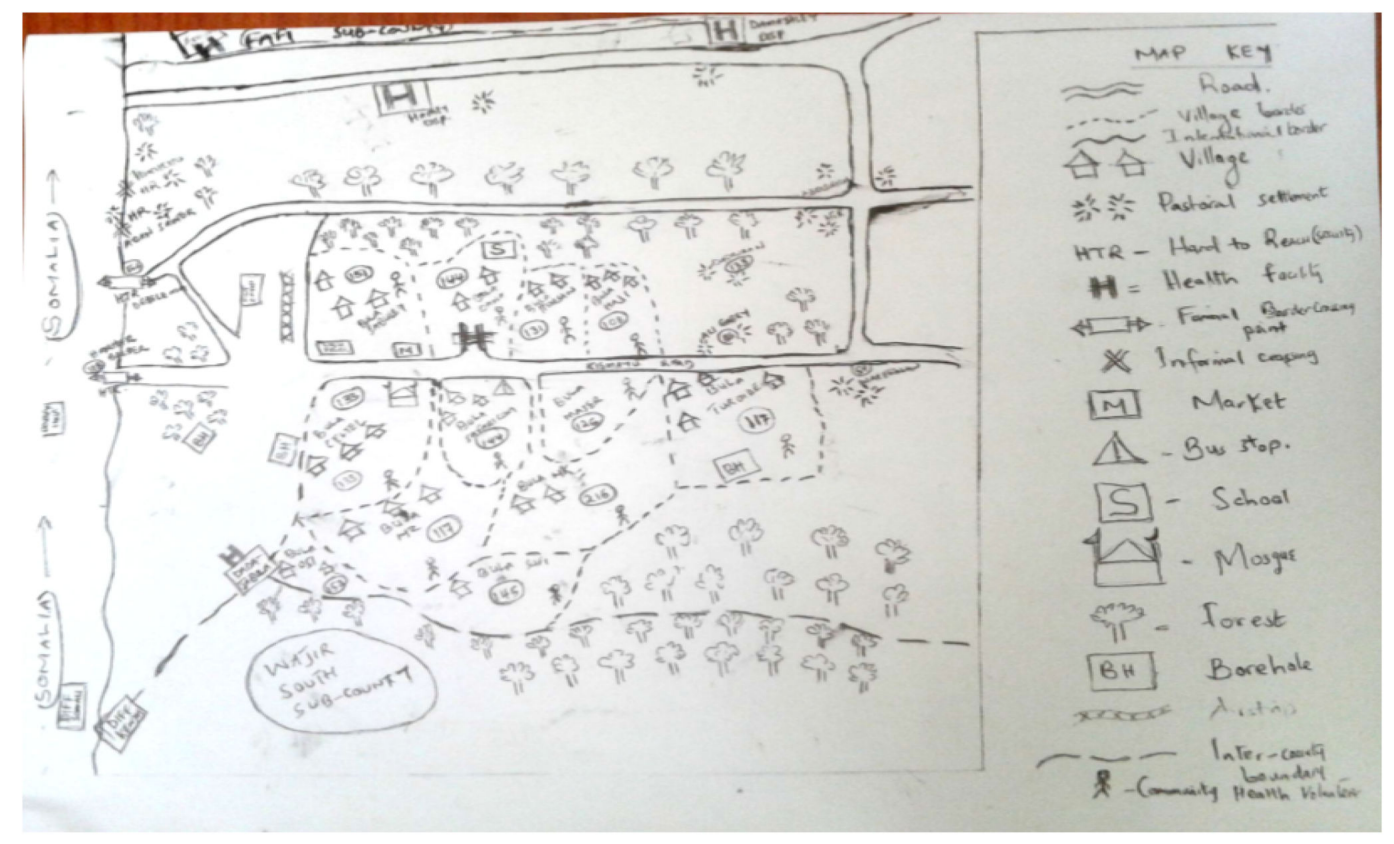
An example of a Sketch map for micro-planning from Dadaab sub-County.

The total number of settlements or nomadic hamlets listed in the two sub-Counties during that process was 79 (Fafi 44, Dadaab 35). A movement tracking form was created containing necessary details for polio campaign planning such as where settlements are located, who are the heads of the families in these settlements, the > 5 population in the settlements, details of the contacts persons for each settlement and the number of children vaccinated in the last SIA (Table 2). Description of special features and landmarks for each settlement indicated the settlements were concentrated around waterpoints in both sub-Counties. The settlements were then put on a sketch map of the sub-County, for example (Figure 1) for Dadaab sub-County to aid both the teams micro-planning for upcoming SIAs and those selected to reach nomadic settlements from the local health facility.

**Figure 2:**
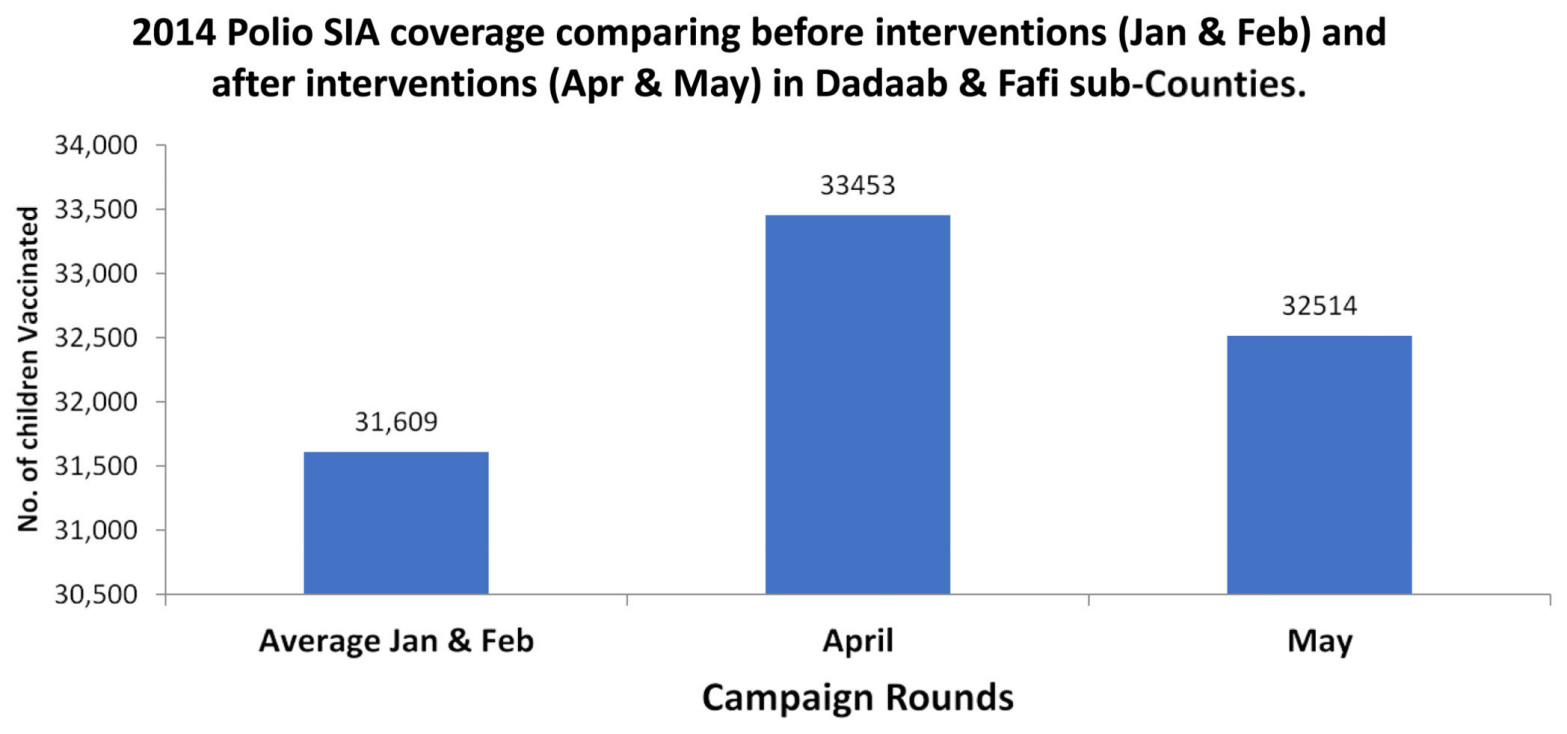
Number of children vaccinated per round Dadaab & Fafi sub –Counties in 2014.

**Figure 3:**
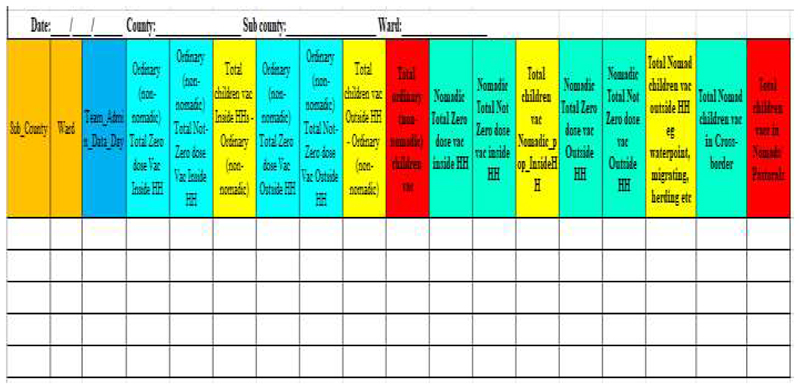
Summary created to disaggregate campaign coverage data; Non-Nomadic and Nomadic children.

**Figure 4:**
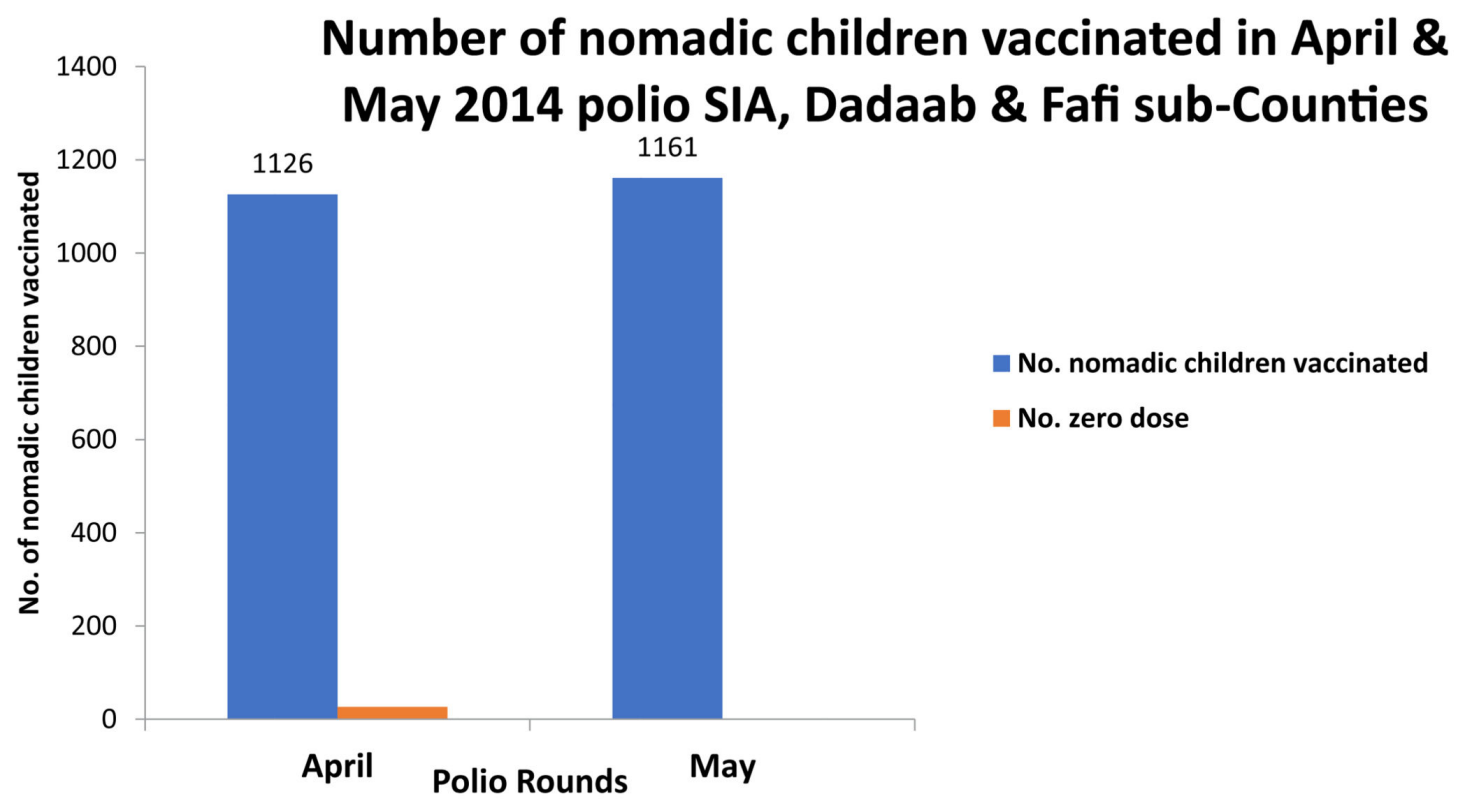
Total and number of zero dose children reached in nomadic settlements per round 2014.

Initial vaccination responses to the outbreak followed traditional practice in the county in which focus was on reaching children in documented settlements. The first round of polio vaccination campaign using the new initiatives was implemented in April 2014. The total number of children (nomadic and urban) reached in both Sub-Counties in this round was 33,453, while the average reached in the preceding two rounds before the initiatives were 31,609. In May 2014, another round of campaign using the new initiatives was conducted (Figure 2). Coverage data was disaggregated for the first time to show the total number of nomadic and non-nomadic children reached per round of vaccination campaign. A summary sheet was designed for this purpose (Figure 3). The number of children from nomadic settlements reached in the two rounds, April and May 2014 were 1126 and 1161 respectively (Figure 4). The number of zero dose children recorded was higher in April 2014, during the first round.

**Figure 5:**
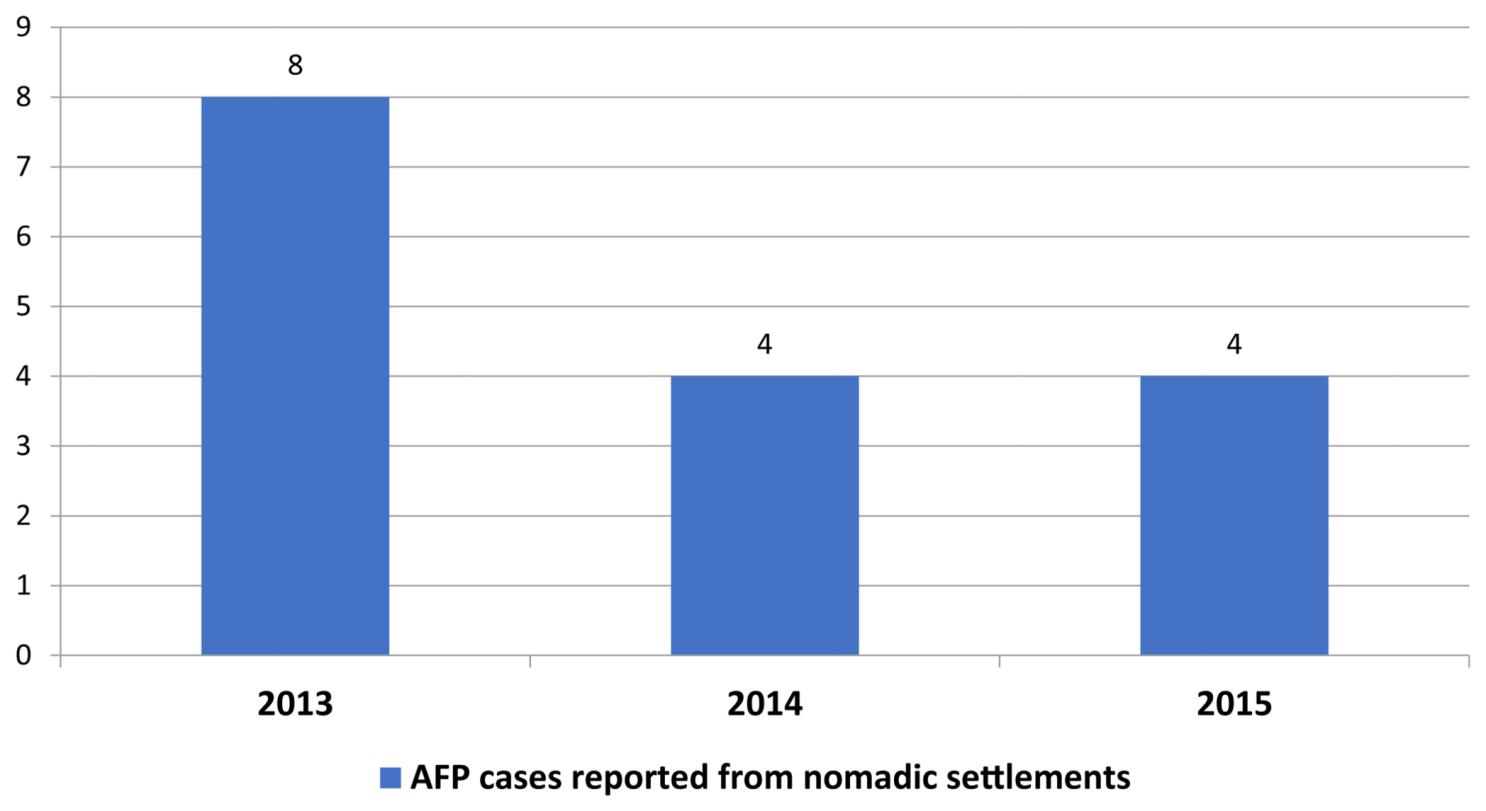
AFP cases reported from nomadic settlements in Dadaab & Fafi sub-Counties 2013 – 2015.

**Figure 6:**
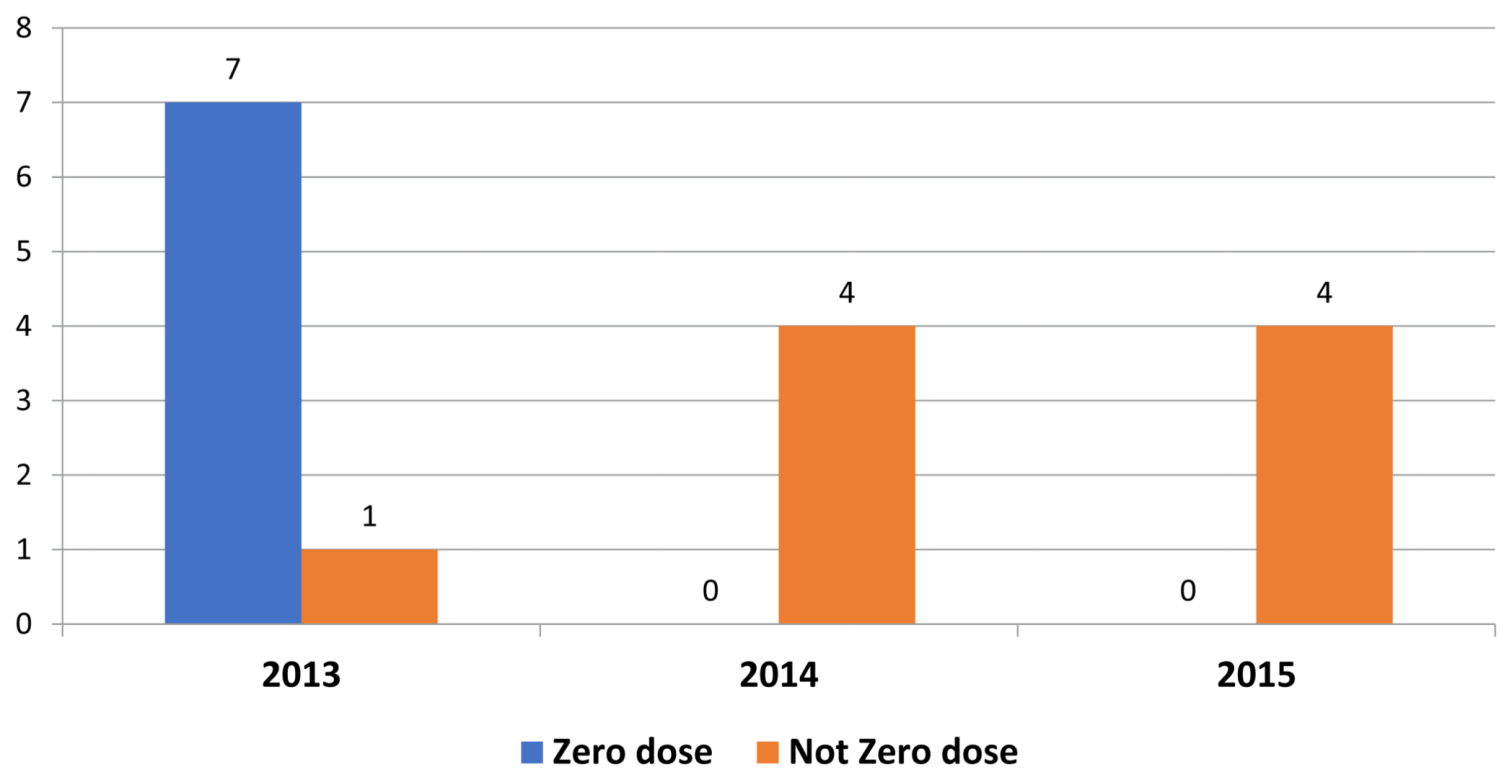
Vaccination status of AFP cases from nomadic settlements in 2013-2015

Regarding reporting of suspected AFP cases from the nomadic settlements, eight cases were reported from both sub-Counties in 2013. Four cases each were reported in 2014 and 2015 from these settlements (Figure 5). Of the cases reported from nomadic settlements in 2013, seven out of the eight had not received a dose of polio vaccine before, while all the AFP cases reported in 2014 and 2015 from these settlements had received at least a dose of oral polio vaccine (Figure 6).

## Discussion

We found that by using clan elders and other key informants in the nomadic communities, it was possible to establish a list of nomadic settlements and their location in the two sub-Counties. These settlements were then mapped and through a focal person identified for each settlement, a tracking system was established to update their movement regularly for the vaccination campaign micro-planning process.

Secondly, the polio vaccination campaign coverage shows that more children, (both urban and nomadic) were reached in the April and May polio campaigns after the new set of initiatives were implemented in comparison to the average number reached before. As indicated earlier, traditional planning for routine services and SIAs was based on accessible populations living in known villages, market centres and urban areas. By involving them in the planning and implementation of the vaccination campaigns, the nomadic clans in the two sub-Counties felt recognized and comprehended their significance in the global polio eradication initiative. By implementing the new initiatives, the health management teams in the two sub-Counties also realized that their nomadic population could be accessed. The inventorying of the nomadic populations in these sub-Counties provided useful data for relief and other government services such as water development, livestock and veterinary services. Further, for the first time, campaign data for these sub-Counties were disaggregated to show the number of children reached in nomadic settlements. Not only that, reporting of suspected AFP cases from nomadic settlements continued in 2014 and 2015. Further, probably as an indication that more nomadic children were reached in the campaigns using the new strategies, all (100%) of the AFP cases reported from nomadic settlements in 2014 and 2015 had received at least a dose of Polio vaccine.

The introduction of these interventions was not planned as a study but intended to improve coverage among nomadic children and get this population’s participation in AFP Surveillance. When they were first being introduced, vaccinators and supervisors were trained on the tools, and there was more scrutiny of data and data entry regarding vaccination of nomadic children. There was a lot of enthusiasm. Advocacy, communication and social mobilisation activities were intense, and records show a higher number of nomadic children reached.

The limitation in this case and all cases dealing with nomads is that since an accurate size of the nomadic population is difficult to know, no list of settlements can be considered comprehensive and no number of children reached can be said to be total. Furthermore, it was possible that even with a tracking tool as was created here, nomadic families moved to another location, sometimes across the border just before or during a campaign. It was also possible that other public and private institutions working in the livestock, veterinary and water areas may have contributed additional useful information in the preparation of such initiatives. Also, the outbreak response was running for about a year, and seven rounds of campaign and awareness creation had been completed. Success may, therefore, be carried over from these efforts.

In conclusion, our observations were that more nomadic children were reached during polio SIA when; mapping and tracking of the nomadic population were improved, simple curative services were integrated into the vaccination campaign, social mobilisation activities were spearheaded by members of the nomadic communities and where necessary ‘community vaccinators’ were used. The nomadic population can also participate in AFP surveillance when the right community structures are used. In the Somali Region of Ethiopia, it was found that the involvement of nomadic community leaders before, and the integration of livestock vaccination into a polio vaccination campaign and markedly improved coverage^15^. It can, therefore, be said that to achieve any meaningful success in a community level programme, identifying and engaging its key decision makers throughout is vital. A study by UNICEF in Doolo Zone of the Ethiopia’s Somali Region in 2014 concluded that the dissemination of important messages and information is usually initiated and brokered by clan leaders and other community members of influence, including key cattle market traders and these were engaged in communicating polio related messages^11^.

The Kenya Ministry of Health and its partners responded to this polio outbreak according to WHO guidelines. This requires immediate outbreak investigation, vaccination response around the cases, strengthening of AFP surveillance and enhancing routine immunisation^16^. We believe that the successes from the strategies used in response to this outbreak may be improved further and be a starting point to expand efforts to reach nomadic populations with critical health services. The takeaway lesson from this experience is that to maintain momentum in the implementation of such interventions, teams assigned for nomadic settlements need to be retained and retrained before each round and supervision strengthened to ensure proper data entry. As we move to attain polio free certification for polio, it is important to use the lesson learnt from this effort for routine immunisation and other vaccine preventable diseases such as Measles. Health management teams need to work closely with other sectors in order to leverage on their success in reaching all children in the area with vaccines.

## Figures and Tables

**Table 1:** Objectives of the new strategies

	Objectives	Initiative
1.	Improve the mapping of nomadic settlements and tracking of their movement to reach them with vaccine during SIAs.	Establish lists of nomadic settlements in both sub-Counties Identify contact persons for each settlement Create a movement tracking form Train vaccinators assigned to reach nomadic settlements Provide drug kits for teams visiting nomadic hamlets
2.	Sustain the participation of the nomadic population in AFP surveillance by improving the linkage between nomadic settlements and MoH. This is to facilitate reporting of suspected AFP cases and receiving feedback.	Sensitise settlement leaders on suspected AFP case reporting Provide information on locations and contacts of health facilities and CHVs where cases may be reported .

**Table 2:** Nomadic villages/settlements list and Tracking form.

	Nomadic Setdement(Vill age/mobile populations)	Head of Family	Sub-Location	Sub-county	Under 5 population	Contact person	Phone of contact person	ACSM strategies to be used	Special features of the area (if any)	Nimber vaccinated during the last SIA
1										
2										
3										
4										
5										
6										
7										
8										
3										
10										
11										
12										
13										
14										
15										
16										
17										
18										
